# Ultra-short echo time cardiovascular magnetic resonance of atherosclerotic carotid plaque

**DOI:** 10.1186/1532-429X-12-17

**Published:** 2010-03-26

**Authors:** Cheuk F Chan, Niall G Keenan, Sonia Nielles-Vallespin, Peter Gatehouse, Mary N Sheppard, Joseph J Boyle, Dudley J Pennell, David N Firmin

**Affiliations:** 1 National Heart and Lung Institute, London, UK; 2 Cardiovascular Magnetic Resonance Imaging Department, Royal Brompton Hospital, London, UK; 3 Hammersmith Hospital, Imperial College Health Care NHS Trust, London UK

## Abstract

**Background:**

Multi-contrast weighted cardiovascular magnetic resonance (CMR) allows detailed plaque characterisation and assessment of plaque vulnerability. The aim of this preliminary study was to show the potential of Ultra-short Echo Time (UTE) subtraction MR in detecting calcification.

**Methods:**

14 *ex-vivo *human carotid arteries were scanned using CMR and CT, prior to histological slide preparation. Two images were acquired using a double-echo 3D UTE pulse, one with a long TE and the second with an ultra-short TE, with the same TR. An UTE subtraction (ΔUTE) image containing only ultra-short T2 (and T2*) signals was obtained by post-processing subtraction of the 2 UTE images. The ΔUTE image was compared to the conventional 3D T1-weighted sequence and CT scan of the carotid arteries.

**Results:**

In atheromatous carotid arteries, there was a 71% agreement between the high signal intensity areas on ΔUTE images and CT scan. The same areas were represented as low signal intensity on T1W and areas of void on histology, indicating focal calcification. However, in 15% of all the scans there were some incongruent regions of high intensity on ΔUTE that did not correspond with a high intensity signal on CT, and histology confirmed the absence of calcification.

**Conclusions:**

We have demonstrated that the UTE sequence has potential to identify calcified plaque. Further work is needed to fully understand the UTE findings.

## Background

Atherosclerosis is a manifestation of advancing age. For the majority of people the ischaemic sequelae are quiescent due to outward remodelling of the vessel to compensate for deposition of plaque [1]. Individuals experience symptoms when the degree of vessel stenosis is in excess of 40%. Traditionally, the gold standard in visualising the degree of stenosis is by luminography [2]. However, it is now accepted that lumen size as demonstrated on luminography does not correlate with the degree of plaque burden [3,4] and a greater emphasis is placed on plaque characterisation in determining vulnerability [4,5].

The recent concept of the 'culprit lesion' takes into account plaque composition and the risk of acute events varies according to the composition [6]. Culprit plaques have a thin fibrous cap, a large lipid core (> 40%) rich in cholesterol with areas of inflammation and neovascularisation, as opposed to stable plaques with a thick fibrous cap, a small lipid core and areas of calcification [7,8]. However, it is still unclear what contribution calcification has on the stability of plaques, as some authors believe that the presence of calcification is associated with increased likelihood of rupture [9] whereas others believe that the location and extent of calcification may confer stability [10,11]. It has been suggested that calcification within the lipid core, away from the fibrous cap as opposed to within or in close proximity to the cap may stabilise the plaque [10].

Multi-contrast weighted Cardiovascular Magnetic Resonance (CMR) together with Late Gadolinium Enhancement (LGE) has been used to characterise plaque [12]. These studies have been validated against histology and demonstrate a high sensitivity and specificity for the different plaque components [13-15]. Although CMR is able to detect areas of calcification which appear as low intensity areas, it is reported that increased susceptibility at higher field strengths may affect the detection and any subsequent quantification [16].

To date, there has been limited experience in using UTE to look at vascular calcification; the technique may offer an alternative approach to conventional CMR in risk stratification. In this very preliminary work, we show for the first time how Ultra-short Echo Time (UTE) subtraction imaging of the ultra-short T2 components has the potential to detect calcification.

## Methods

**Figure 1:**
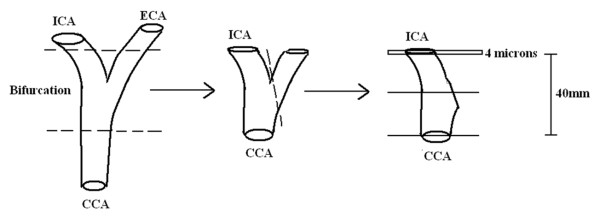
**Diagramatic representation of how each carotid artery was prepared ready for histological slide preparation**. CCA = Common carotid artery, ICA = Internal carotid artery, ECA = External carotid artery. The - - - line represents where the arteries were trimmed.

With full ethical permission from the local ethics committee, and written informed consent, 14 carotid arteries were harvested from 7 individuals at necropsy. There were 4 males and 3 females with a mean age of 64.5 years (range 22-85 years). A short length of artery centred on the carotid bulb was excised. After washing and the removal of adherent soft tissue, the arteries were fixed in 10% formaldehyde for a minimum of 24 hours. For this study, the sections of external carotid arteries were removed so only the internal and common carotid artery remained. The arteries were then trimmed to a standard length with at least 2 cm above and below the carotid bifurcation (figure 1). The samples were scanned in a standardised manner using MR and CT on separate occasions prior to histological slide preparation. For both imaging modalities, the arteries were suspended in a sealed, air-tight container using a 2 mm diameter fibre-optic cable inserted down the common carotid and internal carotid artery. The ends of the cable were secured to the container to prevent movement.

### Magnetic Resonance

**Figure 2:**
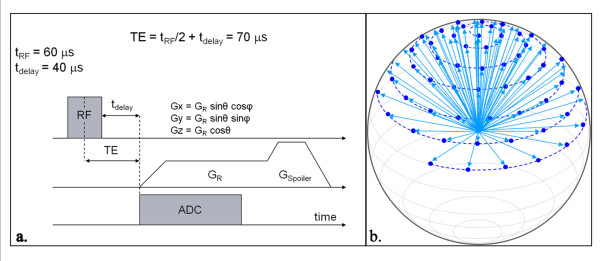
**An illustration of a typical UTE sequence (a) used for this study with 3D radial k-space sampling (b)**. RF = radiofrequency pulse, TE = time to echo, Analogue- to- digital converter (ADC), G_x, y, z _= orthogonal gradients and G_R _and G_Spoiler _= readout and spoiler gradients, respectively. Adapted from Nielles-Vallespin: Proceedings of the 15^th ^Annual meeting of ISMRM 2007 [17].

A 1.5T MR scanner (Siemens Avanto, Erlangen, Germany) and a small 2 cm loop surface coil were used to scan the arteries. Each carotid artery was scanned using a conventional T1W sequence and twice with a radial k-space sampling UTE sequence with 2 different echo times (TE = 2.0 ms and 0.07 ms). The long TE images were subtracted from the UTE images in order to obtain a difference (ΔUTE) image containing only ultra-short T2 signal, which was used for correlation with a CT scan. The measurement parameters for the T1W 3D TSE acquisition were FOV 50 × 20 mm, TR/TE = 1000/15 ms, flip angle 90/180°, readout 256 matrix by 105 phase encode steps, pixel size 0.2 × 0.2 × 1 mm slice thickness, ETL 7, bandwidth 130 Hz/pixel, 1 average. The time taken to acquire the 3D T1W image was ~13 minutes. Two measurements were performed using the 3D UTE acquisition; one with a long TE of 2.0 ms and another with an ultra-short TE of 0.07 ms, both at a TR of 20 ms, FOV 100 × 100 × 100 mm, interpolated pixel size: 0.39 × 0.39 × 0.39 mm, flip angle 30°, reconstructed matrix size 256 × 256 × 256 based on 16384 radial readouts (of 128 samples each at 4.2 μs per sample) distributed over all angles outward from central k-space to the surface of a sphere (figure 2). In order to achieve the shortest possible TE, data acquisition starts during ramp-up time of the readout gradient. The acquisition time for both the short and long TE images was ~6 minutes 30 seconds. T1W and UTE images were obtained sequentially in a single scanning session.

### Computerised Tomography

The arteries were scanned on a Siemens Sensation 64-slice clinical CT scanner as a reference for areas of calcification. Multi-slice CT datasets were acquired with a high resolution setting designed for assessment of coronary artery calcification. Acquisitions were performed at 100 kV, 25 mA, feed per rotation 3.8 cm, matrix 512 × 512 and isotropic voxel size 0.6 × 0.6 × 0.6 mm^3^. Images were reconstructed to give a slice thickness of 1.0 mm.

### Histological slide preparation

The arteries were decalcified in 10% ethylene-diamine-tetra-acetic acid (EDTA) for a period of 9 days. They were then embedded in paraffin wax and sectioned every 1 mm along the vessel with a section thickness of 4 μm. Each section was stained using Haematoxylin and Eosin (H&E) and slides were prepared. The H&E staining gives an overview of the architecture of the vessel and the plaque, including inflammatory cells. Digital images of the histological slides at ×2 magnification were taken using a 5 megapixel camera attached to a Nikon 80i microscope connected to a PC.

### Image comparison and histological slice matching

The T1W images were each 1 mm thick compared to the ΔUTE images 0.39 mm and the CT images 1.0 mm. For T1W and ΔUTE image comparison, the z-coordinate was used to register the same slice location. Imperfect matches were subjectively assessed and the ΔUTE image which most closely resembled the T1W image was used. For slice comparison with CT images, registration was done by identification of the bifurcation. Finally, the corresponding imaging slices and histological slides were matched using the longitudinal position of each slice. Due to the inevitable tissue disruption and shrinkage during processing, slice matching was also verified by a visual assessment comparing histological sections and CMR images.

The carotid ex-vivo tissue and T1W CMR images used in this preliminary study were acquired for a separate and different MR study. The UTE sequence and CT imaging were added after the initial protocol was finalised. As a consequence, exact registration of the carotid arteries using the different imaging modalities was not possible and any quantitative analysis would be inappropriate and misleading. Therefore, analysis was only done visually and agreement/disagreement values are quoted between the UTE and CT images due to the differences in resolution and slice offset.

## Results

All fourteen carotid arteries were scanned successfully with the T1W, UTE sequences and CT protocol. The CMR and CT images were all interpretable and visually matched with the corresponding histology slide. Due to the fact that the ex vivo tissues were harvested for a different study, only 3 out of the 7 pairs of ex vivo specimens had evidence of atherosclerotic plaque disease. The mean ages of the individuals from whom the harvested tissue showed carotid plaque disease compared to those with no disease was 81 years (range 78- 84 years) and 51 years (range 21- 64 years), respectively.

**Figure 3:**
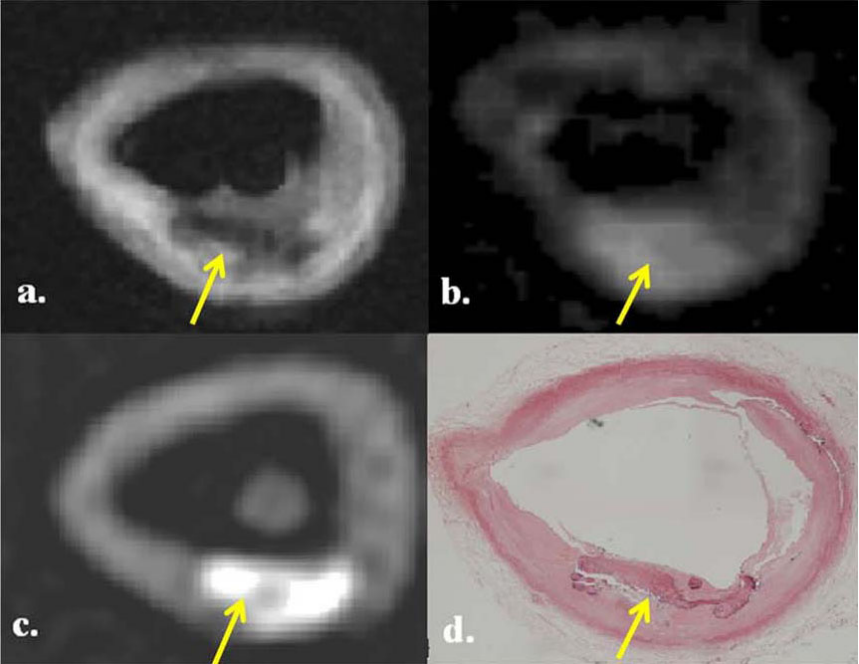
**Multi-modality images of a calcified atheromatous plaque in the common carotid artery (arrowed)**. There is a hypointense area on T1W (a) with corresponding bright area on ΔUTE (b) and CT image (c). Histology section corroborates the presence calcium (d). The * indicates the carbon fibre cable supporting the artery.

We found that on CMR, calcification on T1W images appeared as a dark area and with the ΔUTE images, areas of calcification appeared bright. There was increased signal intensity on CT imaging which corresponded to calcium deposits on histology; these areas of calcification often appeared as a void within the tissue due to the decalcification process. However, some calcium was often still present after processing, and appeared as an acellular dark purple/blue coloured area with well-demarcated edges using Hematoxylin and Eosin (H+E) staining (figure 3).

**Figure 4:**
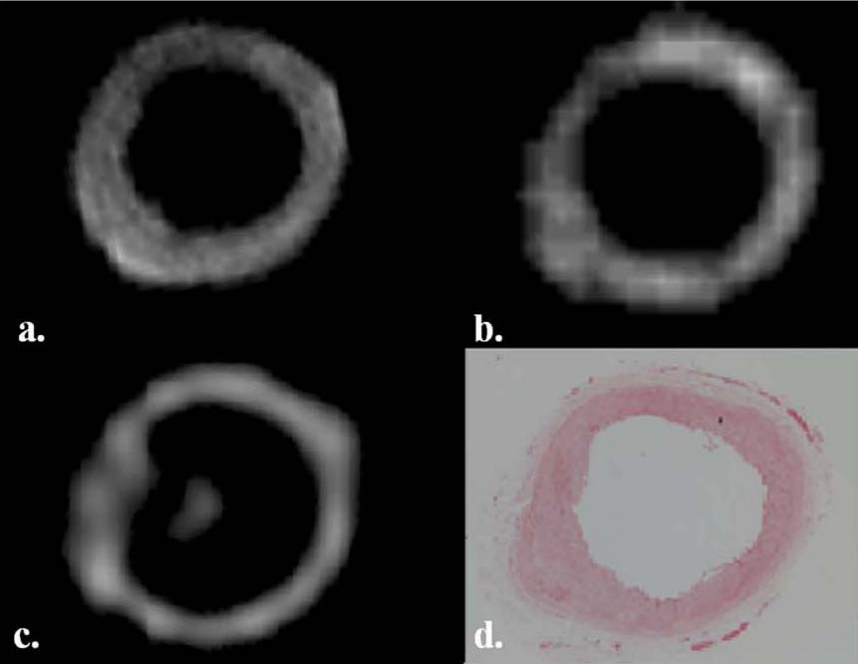
**Images of a 'normal' common carotid artery with no calcification**. (a) T1 weighted image, (b) ΔUTE image, (c) CT image and (d) histology section.

In the 8 diseased carotid artery specimens, there were a total of 96 T1W images which were matched to ΔUTE and CT images, with histology correlation. On the matched corresponding ΔUTE images, we found that there was a 71% agreement (68 out of 96 images) between the bright areas on ΔUTE images which correlated with high signal intensity areas on the CT scans. Therefore, indicating the presence of calcium which was validated using the available histology slides. These bright areas on ΔUTE all had corresponding low intensity areas on T1W (figure 4).

In the "normal" ex-vivo specimens, we found that from a total of 128 matched T1W and ΔUTE images, the majority (> 96%) of the ΔUTE images did not demonstrate any evidence of calcification on the corresponding CT scans. We found no areas of low intensity on T1W for the same image slice. This was corroborated on the available histological sections. Figure 4 shows a series of 4 images comparing matched T1, ΔUTE, CT and histology section of a normal common carotid artery.

**Figure 5:**
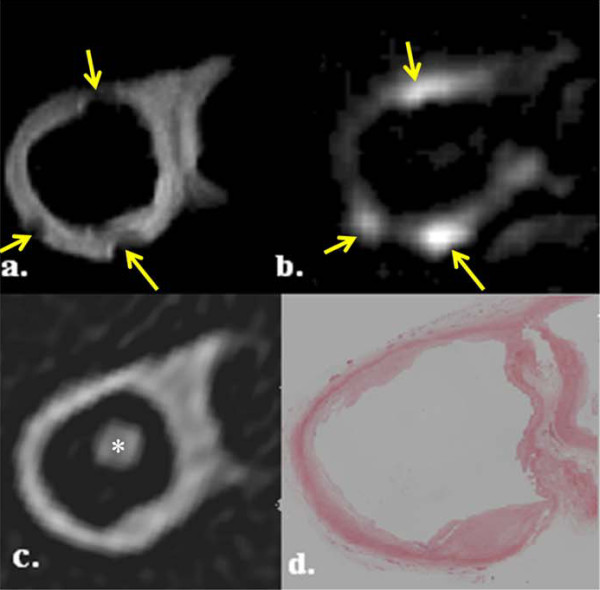
**Likely susceptibility artefact due to remnant formaldehyde droplets on the ex vivi tissue**. Images show an internal carotid artery with incongruent areas (arrowed) on T1W (a) and ΔUTE (b) images compared to the CT image (c) and histology section (d). The * indicates the carbon fibre cable supporting the artery within the internal carotid artery.

However, we also found that on 15% of the total T1W and corresponding ΔUTE images (28 out of the total 224 T1W slices), there were incongruent regions of low signal on T1W together with high signal intensity on ΔUTE images. These did not correspond with zones of calcification on either CT or histology (figure 5). The false positive areas tended to be at the periphery of the vessel or situated within the vessel lumen but not within the wall itself.

## Discussion

The results of this very preliminary imaging study demonstrated that ΔUTE images are able to identify plaque calcification. Our study aimed to corroborate the multi-modality findings with microscopic histology; however we acknowledge that the study lacks quantitative data analysis for the reasons stated above. None-the-less, we believe that these findings have scientific importance in the identification of calcified tissues and should be interpreted in the context of a preliminary study highlighting the potential use of UTE for future studies.

The underlying message of this study is supported by the work by Nielles-Vallespin et al [17]. They showed that non-subtracted UTE images of calcified atherosclerotic aortic plaque appeared as low signal areas which correlated with high signal intensity areas on electron-beam computed tomography (EBCT) and macroscopic ex-vivo calcified tissue. In a study by Herzka et al [18], they looked at UTE pulse sequences without subtraction and correlated the inverted high-resolution UTE colour map with CT to demonstrate calcium deposits within plaque.

The use of CMR in assessing the vessel wall is increasing but it is still predominantly a research tool. Clinically, the commonest non-invasive imaging technique used to assess vessel stenosis is Doppler ultrasound. It can provide an accurate and in-expensive assessment and can potentially be used to characterise plaque structure [19,20]. However, the identification of calcified plaque remains difficult and relies on signal drop-out in otherwise highly echogenic plaque [21]. Recently, the use of EBCT and multi-detector computed tomography (MDCT) has permitted the accurate identification and quantification of calcium in atherosclerotic plaques [22,23]. Although both these techniques can provide information on plaque constituents, are fast, and are able to demonstrate calcification, CT exposes the patient to high doses of ionising radiation and ultrasound is limited by reproducibility issues and inter-observer variability [24]. Neither ultrasound nor CT can offer optimal tissue characterisation.

Atherosclerotic plaque is comprised of various tissue constituents including lipid, fibrous tissue, thrombus and calcification. The classification of plaque is far from simple and the difference between the types of plaque is related to the presence of each component [25]. CMR is able to accurately distinguish between the distinct plaque components by exploiting the different tissue relaxation times using multi-weighted contrast sequences [12,26,27]. Studies conducted have validated the classification of in vivo plaque with ex vivo tissue [28]. However, the presence of calcium within plaque appears as a dark region on standard CMR sequences due to the local dephasing of the water molecules. Consequently, due to the short T2 components of calcium and other tissue components, such as iron, the degree of calcification tends to be over-estimated and, as a result, the clinical risk assessment can be misleading [29-31]. For this reason, other CMR sequences are needed to characterise calcium in plaque.

It is recognised that calcified tissues, tendons and cortical bone, are composed of predominantly ultra-short T2 components [32,33]. With the use of conventional clinical imaging echo times (TE = 15 ms and 75 ms, T1W and T2W, respectively), the MR signal from these tissues has decayed significantly within the TE such that an image with little or no signal from such tissues is produced. A method of distinguishing between those components with ultra-short T2 is by using Ultra-short Echo Time (UTE) which allows detection of the ultra-short T2 components before they have decayed both in tissues with a majority and minority of short T2 components. The typical TE used in UTE sequences are in the range of 50-250 μs, which are ultra-short in comparison with the T2 of calcified tissues (range 13-18 ms) [34]. In clinical practice, such sequences have been used to image cerebral parenchymal tissues [35], knee [36] and ankle [37] ligamentous tissues.

**Figure 6:**
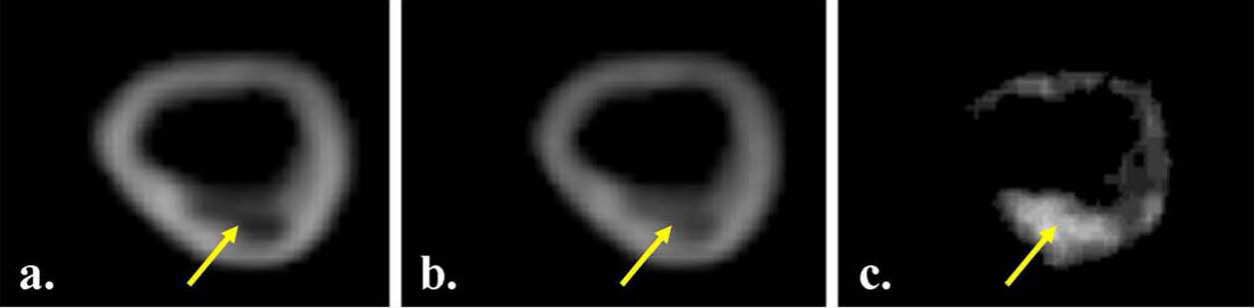
**UTE images acquired with the long TE = 2.0 ms (fig. a) and short TE = 0.07 ms (b) with the resultant subtracted image (c)**.

In this study, by obtaining an UTE image with a long TE (2.0 ms) and fixed TR, the calcified areas appear as low intensity in comparison with the surrounding tissues. The second UTE image with an ultra-short TE (0.07 ms) contains higher intensity signals from those tissues with an ultra-short TE component, such as calcium. Thus, by subtracting the 2 UTE images a resultant image (ΔUTE) with high signal intensity areas corresponding to regions where there was a predominance of ultra-short T2 components is produced (figure 6). As the surrounding tissue would appear to have a relatively low intensity, the resultant image would only depict the calcified tissues and could be a more accurate way of analysing calcification. This process is similar to the theory used in Digital Subtraction Angiography.

However, there were instances (as depicted in figure 5) when T1W and ΔUTE images were suggestive of calcium deposition but there was no evidence of this on the CT scan or on histology. The explanation for this finding is not clear. There are only a few causes of low signal intensity on T1 images; tissues with low proton density, very long T1 or ultra-short T2. On ΔUTE images, it is acknowledged that along with calcified tissues, collagen, tendons and cortical bone, composed predominantly of short T2 components appear as bright areas [32,34]. This finding is likely to be due to susceptibility artefacts, such as from diamagnetic droplets of formaldehyde solution on the surface of the tissue. These would not have been present on the CT data, and not on histology.

### Study limitations

The CMR and CT scans of the ex vivo specimens were undertaken on different days with removal and replacement of the specimens from the holder. There was no regimented way of ensuring the ex vivo specimens were stretched to the length each time. This meant that we encountered some difficulty in co-registering the exact location of the images. In part because of this and the fact that different image types had different in-plane spatial resolution and slice thicknesses, we could only perform visual analysis in the comparison between the T1W, ΔUTE and CT data. Reporting quantitative data in this preliminary study would be misleading. In addition, the ex vivo tissue was stored in formaldehyde solution which may have given rise to susceptibility artefacts from residual droplets on the tissue. For any future work, the tissues could be fixed in Agarose gel with all the air bubbles and formaldehyde solution removed. In order to examine other constituents of the vessel wall fully, further tissue characterisation using T2 weighted imaging would have to have been employed.

The 3D UTE sequence we have used in this preliminary study has been done on ex vivo tissues. The total time taken to acquire the images is 13 minutes; this is very long in comparison to the other 3D carotid sequences [38]. Together with the problems surrounding current 3D methods such as bulk motion artefacts may restrict the technique to ex vivo research arena.

## Conclusion

We have shown that ΔUTE images can potentially be used in conjunction with conventional imaging sequences to identify calcification allowing for further risk stratification and guidance of treatment. Although there are no quantitative data in this study, we believe that it provides a useful platform for further investigation.

## Competing interests

CFC, NGK, SNV, MS, JJB, DNF- The authors declare that they have no competing interests.

DJP- Consultancy Siemens, Novartis Apotex; Research Support Siemens, and Novartis, Director Cardiovascular Imaging Solutions

## Authors' contributions

CC- main manuscript author, coordination of study, acquisition of data, data analysis. NGK -patient recruitment and coordination of study, helped to draft the manuscript, data analysis. SNV- helped to draft the manuscript, data analysis. MNS, JJB- contributed to manuscript, acquisition of data. DJP- participated in the study design and coordination, helped to draft the manuscript. DNF-conceived the study, participated in the study design and coordination, helped to draft the manuscript.
